# Reproductive health services for refugees by refugees in Guinea I: family planning

**DOI:** 10.1186/1752-1505-2-12

**Published:** 2008-10-16

**Authors:** Natasha Howard, Sarah Kollie, Yaya Souare, Anna von Roenne, David Blankhart, Claire Newey, Mark I Chen, Matthias Borchert

**Affiliations:** 1 London School of Hygiene and Tropical Medicine (LSHTM), Keppel Street, London, UK; 2 Reproductive Health Group (RHG), Guéckédou, Guinea; 3 Gesellschaft für Technische Zusammenarbeit (GTZ) GmbH, 65726 Eschborn, Germany

## Abstract

**Background:**

Comprehensive studies of family planning (FP) in refugee camps are relatively uncommon. This paper examines gender and age differences in family planning knowledge, attitudes, and practices among Sierra Leonean and Liberian refugees living in Guinea.

**Methods:**

In 1999, a cross-sectional survey was conducted of 889 reproductive-age men and women refugees from 48 camps served by the refugee-organised *Reproductive Health Group *(*RHG*). Sampling was multi-stage with data collected for socio-demographics, family planning, sexual health, and antenatal care. Statistics were calculated for selected indicators.

**Results:**

Women knew more about FP, although men's education reduced this difference. RHG facilitators were the primary source of reproductive health information for all respondents. However, more men then women obtained information from non-health sources, such as friends and media. Approval of FP was high, significantly higher in women than in men (90% vs. 70%). However, more than 40% reported not having discussed FP with their partner. Perceived service quality was an important determinant in choosing where to get contraceptives. Contraceptive use in the camps served by RHG was much higher than typical for either refugees' country of origin or the host country (17% vs. 3.9 and 4.1% respectively), but the risk of unwanted pregnancy remained considerable (69%).

**Conclusion:**

This refugee self-help model appeared largely effective and could be considered for reproductive health needs in similar settings. Having any formal education appeared a major determinant of FP knowledge for men, while this was less noticeable for women. Thus, FP communication strategies for refugees should consider gender-specific messages and channels.

## Background

Reproductive health programming is never easy, but provision of effective care to populations affected by conflict and complex emergencies poses special challenges [1,2]. The International Conference on Population and Development (Cairo, 1994) and the Fourth World Conference on Women (Beijing, 1995) marked a policy shift for both reproductive health and refugee health. The definition of reproductive health became more comprehensive, emphasising the reproductive health needs and rights of the underserved, particularly refugees and internally displaced persons (IDPs) [3-5]. However, despite strengthened international interest and policy frameworks, implementation remains difficult and quality research is minimal [2-4,6-11]. Published studies have concentrated on refugees in more developed countries or stable camp settings.

Globally, refugees and IDPs number over 32.9 million [6], and "reproductive health needs do not disappear upon displacement" [4]. Relief efforts have traditionally focused on acute-phase survival, including HIV prevention and basic emergency obstetric care [7]. However, reproductive health spans relief and development and is essential for long-term survival [7,8]. This is particularly true of contraceptive services, whose functioning requires sufficient staff training, counselling skills and supplies, and client trust that service quality is good and supplies will continue. In many camp settings, fertility rates and gender-based violence increase, and maternal and neonatal mortality can be high [7,9]. For example, a World Health Organisation (WHO) study estimated 25–50% of maternal mortality among refugees as due to unsafe abortion, indicating considerable unmet need for contraception [10]. Implementing agencies now recognise the need to provide contraceptive services. However, despite moves to improve provision, barriers to access and acceptability remain [11-14].

### Setting

From 1989 to 2004, conflicts in Liberia and Sierra Leone displaced over 500,000 people into the Forest Region of neighbouring Guinea [15]. While many Liberians returned home following elections in 1997, civil war in Sierra Leone lasted until 2002. Two major refugee influxes in 1991 and 1997–98 challenged the already weakened Guinean health services, still recovering from disastrous economic and political conditions under Sekou Touré. Following the 1986 Bamako Initiative, Guinea's new government initiated major health sector reforms, encouraging non-governmental agencies to support health service development. Guinea's Ministry of Health responded to refugee health needs through the "Programme d'assistance aux réfugiés Libériens et Sierra Léonais" (PARLS), which soon became an integral part of the health system. Refugees received free treatment from Guinean health services, reimbursed by UNHCR on a fee-for-service basis. However, refugees sometimes perceived government reproductive and sexual health services as deficient. For example, Liberian and Sierra Leonean women had access to family planning (FP) in their home countries, but in Guinean health centres such services were only introduced in 1992.

In 1995, a group of refugee midwives and interested women organised the '*Reproductive Health Group' *(RHG) to improve on the local services available to their fellow refugees in Guéckédou and Kissidougou prefectures. RHG was supported by GTZ (German Technical Cooperation) as a non-governmental organisation (NGO) for refugee health by refugees. It was based on the innovative concept of rallying expertise within refugee communities to address their own sexual and reproductive health needs. RHG mobilised refugee expertise by recruiting and seconding refugee nurses and midwives to local Guinean health facilities, and training refugee lay women to provide reproductive health education, referrals, and contraceptives for the refugee community. RHG used drama groups to reach those less likely to access facilities or RHG facilitators, particularly young people and men. RHG achieved good coverage in Guéckédou and Kissidougou camps (e.g. antenatal services covered 56% of reproductive-age women). Table 1 summarises the RHG programme, while details are published elsewhere [14,16].

**Table 1:** RHG model summary

**Staff**	Management: 1 coordinator, 1 deputy coordinator, 1 youth coordinator. Support: 4 supervisors, 1 data officer, 1 finance officer, 1 part-time expatriate advisor (GTZ). Frontline: 36 nurse/midwives, 75 RHG facilitators, 14 youth/drama groups.
**Staff training **	Safe motherhood, FP, syndromic STI management, HIV prevention.

**Organisational development**	RHG management team was coached by GTZ in NGO internal governance issues, human resource management, project management, monitoring and evaluation, health information systems, survey design, implementation and evaluation.

**Supplies**	Contraceptives: oral, injectible, IUD, condoms, and spermicide supplied through health facilities. STI drugs: antibiotics for refugees from UNHCR, supplied through health facilities. Other: transport (2 pickups, 2 motorbikes), office and audio/visual equipment from GTZ.

**Funding**	Approximately USD 164,000 annually (1999).

**Partners**	GTZ (core funding, organisational development, technical assistance), ARC (training, funding some facilitators), UNHCR (refugee services coordination, reimbursement of Guinean health services for refugees), Guinean MoH (health facilities).

**Activities**	Health service based: Female refugee nurses and midwives, seconded to 28 Guinean health facilities used by refugees, provided services to refugees and Guineans. Community based: RHG facilitators provided information, contraceptives (condoms, spermicide) and referral. Theatre groups and youth clubs provided information and entertainment.

**Goal**	RH professionals and motivated community volunteers enabled to plan, provide and evaluate sexual and reproductive health services for their fellow refugees.

**Impact**	Improved RH service provision in Forest Region, increased contraceptive usage and STI prevention and treatment, and became an important actor in the health sector (RHG represented 'best practice' and 'worthy of study' – WHO consultant [16]).

Source: von Roenne et al [14].

### Objectives

RHG health education and services appeared to reach women effectively. However, workers were concerned about their effectiveness in reaching men and adolescents, which has been found to be problematic elsewhere [12,17-27]. In 1999, a cross-sectional survey was conducted in the refugee population to gather population-level data on reproductive health knowledge, attitudes and practices (KAP) for use in strengthening RHG's implementation. The survey collected data on demographics, family planning, sexually transmitted infections (STIs), HIV, antenatal and obstetric care. Study objectives included the assessment of gender or age differences in reproductive health knowledge, attitudes, and practices, which might warrant different approaches for these target groups. This paper addresses gender and age differences in family planning, while STIs are addressed in the companion paper [28].

## Methods

### Study design

The study this paper is drawn from was a cross-sectional, questionnaire-based interview survey on sexual and reproductive health knowledge, attitudes, and practices. The target population was reproductive-age male and female refugees (15 to 49) from an estimated population of 250,000 living in 48 camps across the Forest Region of Guinea, covered by RHG activities for four years. Two planned follow-up surveys were abandoned due to political changes and camp closures.

Sampling was multi-stage. First, 45 clusters of households were randomly selected from the 48 camps, with probability of selection proportional to camp size. Second, a stratified sample of ten men and ten women per cluster (i.e. one eligible man or woman from each of twenty households) was randomly selected from household lists. Sample size was calculated to detect a difference of 10% versus 20% between strata of equal size with 80% power and 95% confidence interval, accounting for clustering. Participation was voluntary, with no payments other than reimbursement of travel costs made. The study received ethical clearance from the Ministry of Public Health in Guinea and the London School of Hygiene & Tropical Medicine in the UK.

### Data collection

The questionnaire was designed from instruments used in similar settings and piloted in a camp not included in the study. Several sections covered socio-demographic information, family planning, sexual health, and antenatal care (only for female interviewees). The questionnaire was intended for use in English, and if respondents were not sufficiently fluent, the interviewer translated directly into local language. Questions were read verbatim to ensure reliability, and only rephrased if a respondent did not understand. Interviewers were recruited from the refugee community, and were always the same sex as respondents. A four-day training course and instruction manual were given to all interviewers, covering aspects such as privacy, prompting and translations. Data collection and entry were conducted over four weeks in 1999. Three contact attempts were made before classification as absent and replacement with another household or individual. Data collection and entry were completed within the study period. Data was double-entered in Epi-Info™ 6, with range and consistency checks to reduce transposition error.

### Data analysis

Analysis was conducted using Stata^® ^10.0. Family planning outcomes were explored for associations with gender and age, using chi-squared tests and Mantel Haenszel odds ratios as appropriate. Potential confounders were determined based on independent association with exposure and outcome variables (i.e. significant at p < 0.05). Confounders that changed odds ratios by at least 10% were incorporated into logistic regression models, which accounted for clustering using robust standard errors. Effect modifiers were reported individually if they changed the effect of exposure on outcome significantly between strata, as determined by Wald test.

## Results

### Demographics

The response rate exceeded 95% and the final sample analysed was 889 (445 men and 444 women). Household lists indicated the sexes were represented equally in the study population, and weighting was deemed unnecessary despite stratified sampling by sex. About 60% of respondents were under age 30, with women significantly younger than men. Most refugees (97%) were from Sierra Leone, and at the time of interview, more than half had arrived in camp within the past three years (i.e. after 1996). Sixty percent of men, but only 29% of women had received some formal schooling. Almost all (91%) were sexually experienced. Women were more likely to be married, and 32% reported their husband as having more than one wife. Women were significantly younger than men at marriage (mean age 16 years versus 24 years for men) and at first intercourse (mean 17 years versus 19 for men). See Table 2 for more.

**Table 2:** Demographic characteristics stratified by gender

**Variable **	**Category**	**Male (%)**	**Female (%)**	**X^2^p-value**
*All respondents:*		*445 (100)*	*444 (100)*	
Age	15–19	103 (23)	104 (23)	
	20–29	141 (32)	189 (43)	
	30–39	140 (31)	118 (27)	
	40–49	61 (14)	33 (7)	0.001
				
Country of origin	Sierra Leone	436 (98)	432 (97)	
	Liberia	7 (2)	12 (3)	
	Other	2 (0)	0 (0)	0.19
				
Arrival in camp	Before 1996	202 (45)	188 (42)	
	1996 or later	243 (55)	256 (58)	0.36
				
Education	No formal education	181 (41)	316 (71)	
	Some formal education	264 (59)	128 (29)	<0.001
				
Religion	Catholic	82 (18)	88 (20)	
	Protestant	173 (39)	184 (41)	
	Muslim	190 (43)	172 (39)	0.49
				
Age at first penetrative sex	15 years or less	113 (25)	228 (51)	
	16 years or older	269 (61)	185 (42)	
	Unknown	10 (2)	5 (1)	
	Never	53 (12)	26 (6)	<0.001
				
Marital status	Never married	170 (38)	69 (16)	
	Currently married	251 (56)	320 (72)	<0.001
	Widowed/Separated	24 (6)	55 (12)	
				
Risk of unplanned pregnancy*	No	141 (32)	132 (30)	
	Yes	304 (68)	312 (70)	0.53

*Ever married respondents*		*n = 275 (100)*	*n = 375 (100)*	

Polygyny	Respondent or husband has other wife/wives	58 (21)	120 (32)	0.002
Residence of partner	Living together in camp	237 (86)	275 (73)	<0.001
Age at marriage^+^[29]	10 or under	0 (0)	12 (3)	
	11–17	16 (6)	265 (71)	
	18–29	220 (80)	96 (26)	
	30+	39 (14)	1 (0)	<0.001

*Female respondents*		--	*n = 444 (100)*	

Parity	Nulliparous	--	84 (19)	
	Parous	--	360 (81)	--

*Parous female respondents*			*n = 360 (100)*	

Living children (women)	None	--	36 (10)	
	1–3 children living in household	--	258 (72)	
	4–8 children living in household	--	66 (18)	--

* Those considered at risk for unplanned pregnancy were all those who were between 15–45, had a partner living in camp, reported no contraceptive use, and were not trying to have a child. ^+^Based on WHO definition of adolescence (10–18).

### Family planning

Table 3 shows family planning knowledge, attitude and practice variables stratified by gender. Family planning as a concept could be explained by most study participants (male 66%, female 88%, p < 0.001), but about one-third could not identify a contraceptive method (Table 3a). The mean number of modern methods known by women and men were 1.9 and 1.2 respectively, with condoms and pills most recognised. Except for condoms, which more men identified (61% versus 43%), a significantly higher proportion of women identified each contraceptive method (p < 0.001).

**Table 3:** Family planning knowledge, attitudes and reported practices, by gender

**Variables**	**Category **	**Male (%)**	**Female (%)**	**X^2^p-value**
***a) Knowledge***				

*All respondents:*		*n = 445 (100)*	*n = 444 (100)*	

Can explain FP	Yes	294 (66)	389 (88)	
	Not sure	151 (34)	55 (12)	<0.001
				
No. of FP methods known (excluding traditional methods)	None	144 (32)	132 (30)	
	1–2 methods	241 (54)	159 (36)	
	3–5 methods	60 (14)	153 (34)	<0.001
				
Methods identified w/o probing (multiple answers possible)	Condom	269 (60)	192 (43)	<0.001
	Pill	168 (38)	272 (61)	<0.001
	Injection	68 (15)	206 (46)	<0.001
	Spermicide	13 (3)	85 (19)	<0.001
	IUD	19 (4)	77 (17)	<0.001
	Other (i.e. traditional methods)	49 (11)	77 (17)	0.23
				
Methods identified with probing (multiple answers possible)	Condom	407 (91)	404 (91)	0.8
	Pill	321 (72)	406 (91)	<0.001
	Injection	250 (56)	373 (84)	<0.001
	Spermicide	100 (22)	211 (48)	<0.001
	IUD	110 (25)	228 (51)	<0.001

*Respondents who explained FP*		*n = 294 (100)*	*n = 389 (100)*	

Key FP information source	RHG facilitators	197 (67)	262 (67)	
	Health workers	55 (19)	95 (24)	
	Friends and family	17 (6)	20 (5)	
	Drama groups	14 (5)	11 (3)	
	Radio	6 (2)	0 (0)	
	Other/Unknown	5 (2)	1 (0)	0.006

***b) Attitude***	***Category ***	***Male***	***Female***	***X***^2^***p-value***

*All respondents*		*n = 445 (100)*	*n = 444 (100)*	

Attitude to couples using FP	Approve	326 (73)	396 (89)	
	Disapprove	95 (21)	40 (9)	
	Don't know	24 (5)	8 (2)	<0.001
				
Attitude to RHG facilitators providing FP info	Approve	334 (75)	405 (91)	
	Disapprove	91 (20)	33 (7)	
	Don't know	20 (5)	6 (2)	<0.001
				
Attitude to FP teaching (to boys)	Before age 15	59 (13)	84 (19)	
	Around age 15	155 (35)	200 (45)	
	Later than age 15	120 (27)	102 (23)	
	Disapprove/Don't know	111 (25)	58 (13)	<0.001
				
Attitude to FP teaching (to girls)	Before age 15	158 (36)	223 (50)	
	Around age 15	111 (25)	117 (26)	
	Later than age 15	66 (15)	50 (11)	
	Disapprove/Don't know	110 (25)	54 (12)	<0.001

*Respondents currently with partner*		*n = 251 (100)*	*n = 320 (100)*	

Partner's attitude to couples using FP	Partner approves	163 (65)	190 (59)	
	Partner disapproves	60 (24)	54 (17)	
	Don't know partner's attitude	28 (11)	76 (24)	<0.001

***c) Practice***	**Category **	**Male**	**Female**	**X^2^p-value**

*All respondents*		*n = 445 (100)*	*n = 444 (100)*	

Reported use of contraception	Never	260 (58)	251 (57)	
	Past	78 (18)	77 (17)	
	Current	107 (24)	116 (26)	0.77
				
Use by respondent/partner (multiple answers possible)	Ever used condoms	164 (37)	41 (9)	<0.001
	Ever used pills	71 (16)	116 (26)	<0.001
	Ever used injections	25 (6)	70 (16)	<0.001
	Ever used spermicides	12 (3)	17 (4)	<0.001
	Ever used IUDs	1 (0)	5 (1)	<0.001
				
Contraceptives currently used (multiple answers possible)	Condoms	85 (19)	14 (3)	<0.001
	Pills	32 (7)	59 (13)	<0.001
	Injections	12 (3)	43 (10)	<0.001
	Spermicide	0 (0)	2 (0)	--
	IUD	0 (0)	0 (0)	--

*Respondents currently with partner*		*n = 251 (100)*	*n = 320 (100)*	

Discussion of FP with partner	Never	108 (43)	143 (45)	
	1–2 times in last 12 months	68 (27)	98 (30)	
	More than twice in last 12 months	75 (30)	79 (25)	0.35

*Current users*		*n= 107 (100)*	*n= 116 (100)*	

Where current users access FP	Health post/Clinic	68 (64)	106 (92)	
	RHG facilitators	30 (28)	5 (4)	
	Any other locations	9 (8)	5 (4)	<0.001
				
Why FP source was chosen (multiple answers)	Quality-more privacy	84 (80)	106 (91)	
	Quality-competent staff	86 (82)	97 (84)	
	Quality-friendly staff	82 (78)	94 (81)	
	Cost-cheaper	75 (71)	99 (85)	
	Convenience-closer to home	73 (70)	71 (61)	
	Quality-better product	65 (62)	75 (65)	
	Convenience-shorter wait	66 (63)	71 (61)	
	Quality-cleaner facility	57 (54)	67 (58)	
	Convenience-use other services	44 (42)	80 (69)	
	Quality-only available there	41 (38)	52 (45)	
	Convenience-opening hours	40 (38)	46 (40)	
	Convenience-closer to work/market	28 (27)	28 (24)	--

*Current non-users*		*n = 338 (100)*	*n = 328 (100)*	

Main reason for non-use of modern contraception	Fertility related*	213 (63)	261 (80)	
	Opposed to use	82 (24)	27 (8)	
	Method related	22 (6)	32 (10)	
	Provider related	12 (4)	7 (2)	
	Lack of knowledge	9 (3)	1 (0)	<0.001
				
Expected future contraceptive use	Never	83 (25)	45 (14)	
	Later/Don't know	206 (61)	240 (73)	
	Within next 12 months	49 (14)	43 (13)	0.001

*Current non-users, opposed to FP use*		*n = 82 (100)*	*n = 27 (100)*	

	Religion opposed (all)	45 (55)	7 (26)	
	*(Muslim)*	41(-)	7(-)	
	Respondent opposed	22 (27)	8 (30)	
	Partner opposed	12 (14)	11 (41)	
	Family opposed	3 (4)	1 (4)	0.02

*abstaining, pregnant, lactating, unable or trying to conceive

Female respondents were almost five times more likely to know about family planning concepts and methods than were male respondents (OR 4.8, robust 95% confidence interval 2.9–7.9, adjusted for age, ever married and education). Among those with formal education women had three times higher odds of knowing what family planning was (OR 3.0, robust 95% confidence interval 1.4–6.3, adjusted for age and ever married). Among those with no formal schooling, this difference rose to over six times greater odds (OR 6.4, 3.6–11.1, adjusted for age and ever married). Although the association was only weakly significant (Wald test p-value = 0.07), this suggests that formal education increased the likelihood that men would know about family planning concepts, reducing the knowledge gap between the genders.

RHG facilitators were cited as the main family planning information source for respondents who knew about family planning (67%), though men and women appeared to access health information differently (p < 0.01). While 91% of women and 86% of men got their health information from RHG staff or health facilities, men were more likely than women to get information from friends, radio, and RHG dramas (15% versus 8%).

More than 70% of men and about 90% of women approved of couples using family planning and of RHG facilitators providing information (Table 3b). However, over 40% of respondents reported never having discussed family planning with their partners. Forty-three percent of respondents considered that girls should receive family planning information before age 15, while only 16% felt this to be appropriate for boys. Women responded significantly more positively to attitude questions than did men (p < 0.001), but almost a quarter of women reported not knowing their partner's attitude to family planning. Among respondents who knew what family planning was, women were more than eight times more likely than men were to approve of couples using contraception (OR 8.7, 3.8–20.0, adjusted for education and partner approval of family planning).

More than half of respondents reported never having used modern contraception, and only a quarter identified themselves as current users (Table 3c). Condoms, oral contraceptives, and injections were the most popular. Over 75% of users obtained contraceptives from health facilities. The three main reasons users chose a particular contraceptive source were related to service quality (i.e. privacy, staff competence, staff friendliness). Both women (80%) and men (63%) reported main reasons for not using contraceptives as 'fertility related' (e.g. abstaining, pregnant, lactating, unable or trying to conceive). Opposition to contraceptive use was reported by 24% of men versus 8% of women (p < 0.001). Of those opposed to using contraception, 55% of men and 26% of women, predominantly Muslims, reported this as due to religion (p = 0.02). Among current non-users, odds of expected future contraceptive use were approximately twice as high for women as men (OR 2.19, 1.4–3.5, adjusted for age, education, and ever married).

Over 90% of respondents knew where to access contraceptives, with women slightly more knowledgeable about were to get the pill. Women and men identified at least one correct source for an average of 3.6 and 2.5 contraceptive methods respectively (i.e. condom, pill, injection, IUD, spermicide).

Knowledge of family planning as a concept and approval of couples using family planning did not differ significantly between respondents who were younger or older than 20 years, though knowledge was slightly better among older respondents (OR 1.7, 0.8–3.7, adjusted for parity and ever having married) [29]. Contraceptive use was more frequent in the older age group (OR = 1.5, 1.3–1.8, adjusted for parity and presence of partner in camp).

Those who reported RHG facilitators as their primary information source had non-significantly higher odds of approving of couples using family planning (OR = 1.8, 0.7–4.2, adjusted for parity) and to be current users of contraception (OR = 1.3, 0.7–2.6, adjusted for parity, education, and partner approval of FP). These respondents also had significantly higher odds of discussing family planning with their partners (OR = 2.2, 1.2–3.8, adjusted for parity and education).

## Discussion

### Implications

Comprehensive studies of reproductive health issues among refugees are still relatively rare. This study enabled insight into the influence of gender on family planning knowledge, attitudes and practices in a camp setting. It supports previous findings that men's education helps to increase family planning awareness and attitudes in the way women's life experience (or parity) appears to do [30-32]. Additional research is necessary on effective ways of targeting men, and improving their reproductive health knowledge, attitudes, and practices in refugee settings.

Findings indicate that RHG clients knew more about family planning, and were more likely to approve of and use contraceptives. The consistency of positive associations between RHG activities and knowledge, attitude and practices for family planning and sexually transmitted disease indicators [28] suggests that RHG's model (i.e. involving refugee women as active members in a refugee self-help organisation that trained and supported them to provide education and contraceptives to their community) was effective and could possibly be replicated in similar conflict-affected settings [1,11,14].

### Limitations

Cross-sectional studies, while enabling exploration of multiple outcomes and exposures, are limited by potential reverse causality as explanatory and outcome variables are measured at the same point in time (e.g. better family planning knowledge may result from attending RHG activities, or RHG attendance might result from better knowledge). Possible reporting and observer bias was minimised through surveyor training and questionnaire piloting. Residual confounding is possible, due to lack of data on certain variables (e.g. socio-economic status, desired family size, gender-based violence), which could influence family planning choices. Chance was reduced using robust standard errors.

### Gender and age differences

Education appears a major determinant of men's family planning knowledge, but not of women's knowledge or attitudes. Women were younger and less well educated, yet knew more about family planning and contraceptives. Women are often seen as primarily responsible for family planning, and targeted by reproductive health programming [17]. Possibly, the skills men develop through education enable them to seek knowledge and develop informed opinions on topics to which they might not be exposed otherwise. Additional education may change men's attitude towards gender relations. They may want women as partners who are "more than mothers" [14]. Thus, women's greater ability to contribute to household income could be a reason why men expressed support of family planning and girls' education. Men prioritised non-healthcare information sources, such as radio and dramas, supporting suggestions that men tend to access family planning information in non-health settings [24-26]. It appeared that women respondents learned about family planning through life experience, as knowledge was not significantly influenced by education or exposure to RHG activities (using "time in camp" as proxy). Parity could be a catalyst, as parous women appeared significantly more knowledgeable about family planning than nulliparous women did (p < 0.001). Parity also meant exposure to RHG information during antenatal services.

High reported approval of family planning (80% of respondents) did not correspond with current usage (25%). Main reasons reported for non-use were fertility related (71%). However, usage was much higher than typical for West Africa. UN estimated use of current modern contraceptives for 16 West African countries was 7.9%, with Sierra Leone and Guinea at 3.9% and 4.1% respectively, while RHG's contraceptive coverage was 17% [14,27]. It is difficult to assess whether use was higher in this population because of greater need or better access, but this relatively high coverage supports the value of RHG's work. Nonetheless, our findings suggest that there was still considerable unmet need for contraception and risk of unplanned pregnancy (69%). Interestingly, only 3.5% of non-users reported the barrier of 'partner opposition' noted in the literature [19,21,33]. It is worth noting that respondents rated quality issues higher than cost or distance when choosing contraceptive services.

Findings indicate that adolescents knew somewhat less about family planning, and sexually active young people were somewhat less likely to use contraception, than adults [29]. However, while results suggest additional attention should be given to adolescent reproductive health, fewer age than gender related disparities were found.

Analysis indicates disparities in family planning knowledge and approval between men and women refugees. Given that refugee men know significantly less about family planning and accessed information through peer networks and mass media as well as healthcare providers, communication strategies on family planning in refugee settings could have greater reach with gender-specific messages and communication channels [17,18,26]. The international community should support operational research, involving knowledgeable members of the refugee community, on the best methods of supporting men's utilisation of reproductive health and family planning services within their communities [20,22,23].

## Competing interests

The authors declare they have no competing interests.

## Authors' contributions

NH analysed the data and drafted the paper, contributed to data interpretation, and gave final approval of the version for publication. SK and YS contributed to conception and design, acquisition of data, and reviewing the paper. AvR conceived the study, and contributed to design, data interpretation, and reviewing the paper. DB contributed to design, data interpretation, and reviewing the paper. CN contributed to analysis and data interpretation, and drafting the paper. MC contributed to analysis, data interpretation, and critical revision of the paper. MB designed the study, contributed to acquisition, analysis and interpretation of data, and critical revision of the paper. All authors approved the version to be published.
